# Impact of PM2.5 Emitted by Wood Smoke on the Expression of Glucose Transporter 1 (GLUT1) and Sodium-Dependent Vitamin C Transporter 2 (SVCT2) in the Rat Placenta: A Pregestational and Gestational Exposure Study

**DOI:** 10.3390/antiox14091050

**Published:** 2025-08-26

**Authors:** Francisca Villarroel, Eder Ramírez, Nikol Ponce, Francisco Nualart, Paulo Salinas

**Affiliations:** 1 Laboratory of Animal & Experimental Morphology, Institute of Biology, Faculty of Sciences, Pontificia Universidad Católica de Valparaíso, Valparaíso 2362807, Chile; francisca.villarroel@pucv.cl; 2 Laboratory of Neurobiology and Stem Cells NeuroCellT, Department of Cellular Biology, Faculty of Biological Sciences, Universidad de Concepción, Concepción 4070386, Chile; edramirez@udec.cl (E.R.); frnualart@udec.cl (F.N.); 3 PhD Program in Morphological Sciences, Universidad de La Frontera, Temuco 4811230, Chile; nikol.ponce@ufrontera.cl; 4 Center of Excellence in Surgical and Morphological Studies (CEMyQ), Universidad de La Frontera, Temuco 4811230, Chile; 5 Center for Advanced Microscopy CMA BIO-BIO, Universidad de Concepción, Concepción 4070386, Chile

**Keywords:** PM2.5, placental transporters, GLUT1, SVCT2, oxidative stress, air pollution, pregnancy

## Abstract

Fine particulate matter (PM2.5) emitted by wood smoke is a significant environmental pollutant associated with oxidative stress and hypoxia. These conditions can disrupt placental function by altering the expression of key nutrient transporters, such as glucose transporter 1 (GLUT1) and sodium-dependent vitamin C transporter 2 (SVCT2), which are essential for fetal development. This study evaluates the effects of pregestational and gestational exposure to PM2.5 on GLUT1 and SVCT2 expression in the rat placenta. Pregnant Sprague–Dawley rats were exposed to either filtered air (FA) or non-filtered air (NFA) containing PM2.5 from wood combustion in a controlled exposure system. Four experimental groups were established: FA/FA (control), FA/NFA (gestational exposure), NFA/FA (pregestational exposure), and NFA/NFA (continuous exposure). Immunofluorescence and confocal microscopy were used to quantify the expression of GLUT1 and SVCT2 in the placental labyrinth zone. Statistical analyses were performed using Kruskal–Wallis and post hoc Dunn’s test (*p* < 0.05). Gestational exposure to PM2.5 (FA/NFA) significantly reduced GLUT1 and SVCT2 expression, compromising glucose transport and antioxidant protection in the placenta. Pregestational exposure (NFA/FA) induced a compensatory increase in SVCT2 expression, suggesting an adaptive response to oxidative stress. Continuous exposure (NFA/NFA) resulted in GLUT1 redistribution within the syncytiotrophoblast and decreased membrane localization, potentially impairing glucose uptake. PM2.5 exposure disrupts the expression and localization of GLUT1 and SVCT2 in the placenta, with differential effects depending on the timing of exposure. The gestational phase appears to be particularly vulnerable, as reduced GLUT1 and SVCT2 levels may impair fetal nutrition and antioxidant defense. These findings underscore the need for preventive measures to mitigate air pollution-related risks during pregnancy.

## 1. Introduction

Fine particulate matter (PM2.5), due to its small size, can penetrate deeply into the respiratory system and bloodstream, triggering an inflammatory response and hypoxia. This condition increases the production of reactive oxygen species (ROS) and oxidative stress (OS). Such effects are not limited to lung tissues but also extend to the cardiovascular and reproductive systems, impairing fetal development and maternal health [1,2]. Hypoxia-induced OS elevates the risk of various chronic pathologies [3,4,5,6].

In the context of reproductive health, the placenta plays a crucial role by separating maternal and fetal blood through a barrier formed by the fetal capillary endothelium and the placental trophoblast [7]. This barrier regulates the exchange of gases and nutrients between the mother and fetus, ensuring optimal fetal growth. One such nutrient is glucose, whose transport is mediated by facilitative glucose transporters (GLUTs), with GLUT1 being fundamental for adaptation to hypoxic conditions [8]. Under hypoxic conditions, GLUT1 expression is upregulated, and its translocation to the cell surface is associated with a decrease in intracellular adenosine triphosphate (ATP) levels, reflecting an adaptive response to the low-oxygen environment aimed at ensuring an adequate energy supply to the fetus. This ATP reduction is a key factor triggering the movement of GLUT1 to the cell membrane to enhance glucose uptake, thereby securing ATP production and cell survival under low oxygen availability [9]. However, studies suggest that chronic hypoxia may negatively disrupt this process, affecting fetal growth [10].

During pregnancy, oxidative stress (OS) increases considerably due to a systemic inflammatory response that leads to enhanced production of reactive oxygen species (ROS) and reactive nitrogen species (RNS) within the circulatory system [2,11,12]. The escalation of OS can trigger a range of adverse outcomes, including placental dysfunction and pregnancy complications such as preeclampsia, embryonic resorption, recurrent pregnancy loss, fetal developmental abnormalities, intrauterine growth restriction, and in severe cases, fetal death. Moreover, OS is associated with common disorders such as gestational diabetes and has the potential to impact long-term fetal metabolic programming [5,13,14,15,16]. OS has also been identified as a key factor in placental damage, exacerbating hypoxia and contributing to fetal growth retardation [17,18,19,20]. Vitamin C is essential for counteracting the rise in OS during pregnancy due to its antioxidant properties [21]. In the placenta, vitamin C enters the trophoblast through Sodium-dependent Vitamin C Transporter 2 (SVCT2) [22], which mediates the uptake of the reduced form of vitamin C, known as ascorbic acid (AA) [23]. It has also been proposed that GLUT transporters, certain isoforms of which facilitate the transport of the oxidized form of vitamin C, dehydroascorbic acid (DHA), may participate at the placental level [22,23]. This suggests a potential functional interaction between SVCT2 and GLUT1 in the placenta to enhance the efficiency of vitamin C transport in this organ [24,25]. Vitamin C deficiency increases placental OS, thereby contributing to cellular damage and gestational complications [19,26]. Although maternal–fetal transport of vitamin C is critical, its regulation under OS conditions induced by air pollution remains poorly understood. Simultaneous alterations in GLUT1 and SVCT2, such as those induced by PM2.5 exposure from wood smoke, could synergistically compromise fetal energy supply and antioxidant defense, increasing susceptibility to oxidative stress and limiting the placenta’s adaptive capacity [1].

In Chile, the conurbation of the city of Temuco–Padre Las Casas is characterized by persistently elevated levels of fine particulate matter (PM2.5), exceeding thresholds considered safe for human health. During the winter season, daily PM2.5 concentrations frequently surpass 120 µg/m^3^ [27,28]. This phenomenon is closely linked to the use of firewood for residential heating, which accounts for more than 90% of the PM2.5 emissions polluting the air in Chile [29,30]. In 2021, the region recorded a peak concentration of 157 µg/m^3^ and documented 12 episodes in which concentrations exceeded 100 µg/m^3^. The annual average concentration for that year was 26.8 µg/m^3^ (±30.6; CV: 117%), a value five times higher than the limit recommended by the World Health Organization, which is 5 µg/m^3^.

Previous studies by our group demonstrated that exposure to PM2.5 during gestation is associated with placental hypoxia and fetal growth restriction [1,30]. These effects were also linked to increased expression of angiogenic factors, suggesting a placental adaptation to the hypoxic environment. However, the cellular and molecular mechanisms underlying exposure to wood smoke-derived PM2.5—particularly those related to the transport of essential nutrients and antioxidants such as glucose and vitamin C under hypoxic conditions—remain poorly explored and constitute the focus of the present study. Given the adverse impact of wood smoke-derived PM2.5 on reproductive health—including hypoxia, oxidative stress, and alterations in fetal growth—there is a pressing need to understand how this exposure affects placental function, particularly the transport of essential nutrients such as glucose and vitamin C. The transporters GLUT1 and SVCT2 play a key role in the uptake of these nutrients in the placenta; however, their regulation under environmental pollution conditions, such as exposure to PM2.5, remains poorly explored. Therefore, the objective of this study was to evaluate the impact of pregestational and gestational exposure to wood smoke-derived PM2.5 on the expression and distribution of GLUT1 and SVCT2 transporters in the placenta of Sprague–Dawley rats. The following research question is posed: How does pregestational and gestational exposure to wood smoke-derived PM2.5 affect the expression and distribution of GLUT1 and SVCT2 in the rat placenta and what are the implications of this regulation for glucose and vitamin C uptake under chronic hypoxic conditions? We hypothesized that gestational or combined (pregestational and gestational) exposure to wood smoke-derived PM2.5 reduces expression and alters the subcellular distribution of GLUT1 and SVCT2 transporters, thereby compromising glucose and vitamin C uptake, whereas exclusive pregestational exposure induces a compensatory increase in their expression as an adaptation to chronic hypoxia and oxidative stress, with differential effects depending on the placental cell type. This study demonstrates that gestational or combined exposure to wood smoke-derived PM2.5 reduces the expression and alters the subcellular distribution of GLUT1 and SVCT2 in cytotrophoblasts, syncytiotrophoblasts, and endothelial cells of the rat placenta, likely due to oxidative stress and hypoxia, while exclusive pregestational exposure induces a compensatory increase in their expression, suggesting differential regulation that affects glucose and vitamin C uptake depending on the timing of exposure. These findings provide a foundation for understanding the underlying mechanisms of the adverse effects of PM2.5 on fetal development.

## 2. Materials and Methods

### 2.1. Exposure Site

The experimental exposures were conducted in the city of Temuco, located in southern Chile (38°44′39.59″ S, 72°37′39.07″ W). This urban area has been reported as the sixth most polluted in the country [31], with domestic wood-burning heaters historically representing the main contributor to airborne particulate matter [1,29,32,33,34,35]. The study took place during the austral winter, from 15 June to 30 September 2021. Throughout this period, the animals were maintained under stable housing conditions (temperature 20–25 °C, 12:12 h light–dark cycle) and provided with unrestricted access to food and water. No additional industrial emission sources were identified in the vicinity during the experimental phase.

### 2.2. Exposure Conditions

**Figure 1:**
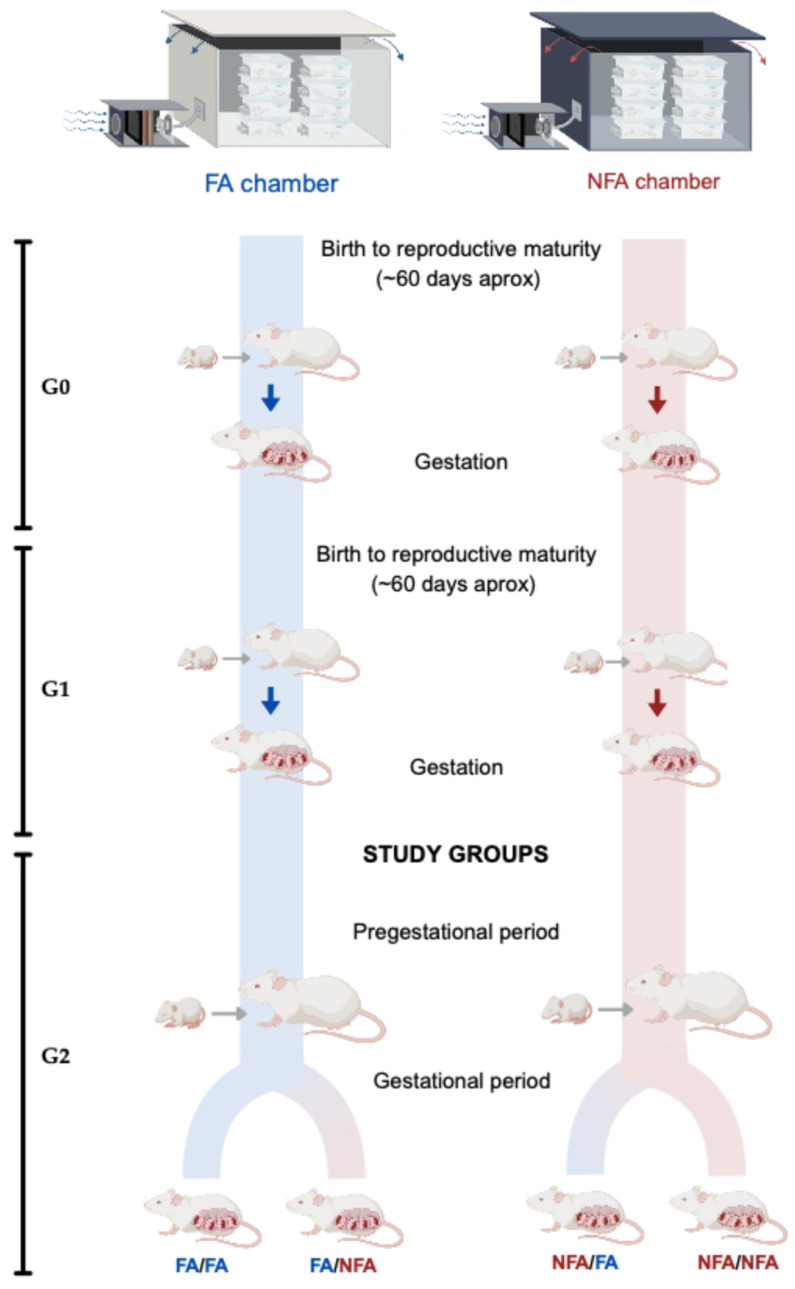
Experimental design, chambers, and exposure groups. This study evaluated the association between PM2.5 exposure and morphofunctional features of the rat placental disc using a crossover experimental design. Two generations (G1 and G2) of Sprague–Dawley rats were included, with the analysis centered on G2 animals to examine the impact of pre-gestational exposure (from birth until reproductive maturity) and gestational exposure (21 days post-fertilization). The G2 cohort (n = 12 per group) was allocated into four experimental groups according to air quality conditions during both stages: FA/FA, FA/NFA, NFA/FA, and NFA/NFA (FA = filtered air, NFA = non-filtered air). Offspring from the G1 generation were raised until reproductive maturity and subsequently assigned to groups based on their combined pregestational and gestational exposure histories.

Two independent but adjacent exposure chambers were built in the courtyard of the Faculty of Medicine, University of La Frontera, situated in Temuco’s downtown area, approximately 500 m from the local air quality monitoring station (Figure S1A,B; Supplementary Document S1; coordinates: −38.7496844990132, −72.6188400896599). This site is recognized for its persistently elevated air pollution levels [27]. Each chamber had internal dimensions of 2.1 m × 2.0 m × 2.1 m and a ventilation system delivering a total airflow of 20 m^3^ min^−1^, allowing for the housing of up to 50 cages. Air was supplied via an industrial fan (capacity 150 m^3^/h, 16.9 m/h, 230 V; Zepol, S.L., model CBB60N, Barcelona, Spain), ensuring homogeneous distribution before release through an outlet located at the upper section. The chambers operated under normobaric conditions, with internal pressure never exceeding 33 mmH_2_O. Both units were maintained under comparable environmental settings; however, in one chamber the incoming air was filtered using a three-stage system (Figure 1). The first two stages removed large and medium particles by means of metal and pleated elements (24 × 24 × 2 cm) incorporating MERV8 filters, followed by a HEPA PH97 unit capable of eliminating 99.97% of particles > 0.3 µm. The final stage consisted of a Purafil PSA 102 system (500 cfm; Purafil Inc., Doraville, GA, USA) equipped with Purafil Select PK12 filter modules (Purafil Inc., Doraville, GA, USA).

### 2.3. Air Analysis

The concentration of PM2.5 within the exposure chambers was measured on a daily basis using a digital analyzer. Outdoor measurements were obtained with a BAM 1020 beta attenuation monitor (Met One Instruments, Inc., Grant Pass, OR, USA), operating with a carbon-14 beta source (4C) of 60 μCi ± 15 μCi (2.22 MBq). This device included a beta detector based on a photomultiplier tube coupled to an organic plastic scintillator, functioning at an airflow of 16.7 L/min. All PM2.5 results were reported in µg/m^3^. Data were supplied by Algoritmos y Mediciones Ambientales SpA from the Las Encinas Monitoring Station, located approximately 200 m from the experimental chambers, and are publicly accessible through the National Air Quality Information System (https://sinca.mma.gob.cl; accessed on 31 December 2021). Concentrations of NO_2_ and CO gases showed similar values in both chambers, as the filtration system used did not remove these specific pollutants.

### 2.4. Study Design

We investigated the relationship between exposure to PM2.5 and the morphofunctional characteristics of the placenta using a crossover case [36]. Following Chilean Law 20.380 and the Guide for the Care and Use of Laboratory Animals (2011) and approved by the Scientific Ethics Committee of the Universidad de La Frontera (Record 122/20), two generations of rats were exposed to air pollution. In the development of animal procedures, we have rigorously adhered to current legislation and guidelines established for animal research. Furthermore, we have ensured compliance with the ARRIVE guidelines (Animals in Research: Reporting In Vivo Experiments [37]) to ensure transparency and ethics in our animal research. Our study centered on the second-generation (G2) rats to examine the effects of pregestational and gestational exposure to PM2.5, aiming to minimize confounding variables and inherited effects from previous exposures in earlier generations (G0). This methodology enabled us to directly isolate and assess the impact of PM2.5 exposure on the health of the placenta in G2 and the fetal size in G3, thereby offering a more precise comprehension of the health implications associated with such exposure. Four groups of first-time mother G2 rats were continuously exposed from birth until the day of the cesarean section (21 days post-fertilization; 21-dpf). G2 rats were consistently exposed to Filtered Air (FA; n = 24) and Non-Filtered Air (NFA; n = 24) from birth until the first day of pregnancy (pregestational). Subsequently, during the gestational stage, each group was divided into two exposure groups (n = 12) (gestational). The G1 rats came from pregnancies in both chambers. Upon maturing, 24 G1 females were mated, giving rise to the G2 rats. These, upon reaching reproductive maturity (around 60 days), were evaluated using vaginal cytology and observation of preovulatory follicles. The estrous cycle stage was determined with daily vaginal cytology, and to confirm gestation, the presence of a semen plug in the vagina was checked. The sample size was determined based on prior studies from our group evaluating the impact of PM2.5 exposure on placental morphology and fetal development, which reported significant differences in placental oxidative stress and transporter expression using group sizes of 8 to 10 animals [1,30]. To ensure sufficient statistical power to detect biologically meaningful differences in transporter expression (GLUT1 and SVCT2) and cellular localization among exposure groups, we included 12 animals per group, which exceeds the minimum number required to achieve a power of 80% with an alpha level of 0.05 and an effect size of 1.2 (G*Power 3.1, two-tailed Kruskal–Wallis test). This conservative estimate also accounts for potential losses during gestation and sample processing. No formal a priori sample size calculation was conducted, but the group size was based on the principles of reduction and refinement, aligning with the ARRIVE 2.0 guidelines. Only healthy, nulliparous female Sprague–Dawley rats from the second generation (G2), aged approximately 60 days and with regular estrous cycles determined by daily vaginal cytology, were included in the study. Females were selected based on the presence of preovulatory follicles and confirmed gestation through detection of a vaginal semen plug. Animals displaying irregular estrous cycles, clinical signs of illness, or failure to conceive after two mating attempts were excluded from the experiment. No animals or data points were excluded post hoc from the analyses. All experimental units (n = 48) reached the defined endpoint (21 days post-fertilization), and complete datasets were obtained for each case. After confirming pregnancy, the G2 females were randomly assigned to one of the four experimental groups (FA/FA, FA/NFA, NFA/FA, NFA/NFA) using a stratified randomization method based on body weight to ensure balanced distribution of physiological conditions across groups. Randomization was performed using a computer-generated list. The allocation sequence was concealed from the experimenters responsible for histological and image analyses, ensuring partial blinding of outcome assessment. Researchers responsible for histological processing, immunofluorescence image acquisition, and quantitative analysis were blinded to the group allocation. Samples were anonymized using coded identifiers until the completion of all image quantifications and statistical analyses. Investigators involved in the animal exposure phase were not blinded due to the logistical requirements of the exposure system.

### 2.5. Groups

Following mating, the G2 females were allocated into four experimental groups: FA/FA (control group; animals reared and completing gestation in a chamber equipped with an air filter), FA/NFA (animals reared in a filtered-air chamber but completing gestation in an unfiltered chamber), NFA/FA (animals reared in an unfiltered chamber but completing gestation in a filtered chamber), and NFA/NFA (animals reared and completing gestation in an unfiltered chamber). All G2 females selected for the study had experienced at least one estrous cycle prior to mating (Figure 1).

### 2.6. Outcome Measures

The primary experimental outcome was the expression and subcellular localization of the glucose transporter GLUT1 and the sodium-dependent vitamin C transporter SVCT2 in the placental labyrinth zone at gestational day 21, as indicators of nutrient and antioxidant transport capacity under PM2.5 exposure. These were quantified using immunofluorescence and confocal microscopy. Specific parameters measured included the following: (i) area of positive staining (µm^2^) and (ii) fluorescence intensity (arbitrary units) of GLUT1 and SVCT2 in individual cells. Secondary outcomes included the colocalization of these transporters with the plasma membrane marker wheat germ agglutinin (WGA), nuclear area (µm^2^), and Manders’ overlap coefficients for transporter–membrane interactions. Cell types analyzed included cytotrophoblasts, syncytiotrophoblasts, and endothelial cells, which were identified and segmented using morphometric criteria in StrataQuest software (version 7.1.1.138; TissueGnostics GmbH, Vienna, Austria).

### 2.7. Immunofluorescence and Image Acquisition

To examine the localization of SVCT2 and GLUT1 transporters in placental tissue, an immunohistochemical protocol was applied. Paraffin-embedded placental sections, each cut at 7 µm thickness, were processed by deparaffinization in xylene, followed by sequential rehydration through graded ethanol concentrations. Primary antibody incubation was performed using anti-GLUT1 (1:400; EMD Millipore, Burlington, MA, USA; #07-401), anti-SVCT2 (1:1000; Santa Cruz Biotechnology, Dallas, TX, USA; SC-9927), and wheat germ agglutinin (WGA) conjugated to Alexa Fluor 488 (1:500; Invitrogen, Carlsbad, CA, USA; #11261). Antibodies were prepared in a Tris/HCl buffer (pH 7.8) containing 8.4 mM sodium phosphate, 3.5 mM potassium phosphate, 120 mM NaCl, and 1% BSA. Sections were incubated with this mixture overnight at 20 °C in a humidified chamber. After thorough washing, samples were exposed for 2 h at room temperature to Cy2-, Cy3-, or Cy5-conjugated secondary antibodies (1:200; Jackson ImmunoResearch, West Grove, PA, USA). Nuclear staining was achieved using DAPI (1:1000). Immunofluorescence imaging for SVCT2, GLUT1, and WGA was performed using the Zeiss LSM 780 confocal microscope (Carl Zeiss Microscopy GmbH, Jena, Germany) at the Center for Advanced Microscopy CMA Bio-Bio (www.cmabiobio.cl).

### 2.8. Cell Analysis and Classification

To identify cellular populations (cytotrophoblast (CT), syncytiotrophoblast (ST), and endothelial cells (ECs)) and analyze the expression of WGA, SVCT2, and GLUT1 in these populations, *.czi* files were used, obtained through high-resolution imaging with a Zeiss LSM 780 confocal microscope. These images were subsequently processed and analyzed using StrataQuest software, version 7.1.1.138 (TissueGnostics GmbH, Vienna, Austria), specifically configured for nuclear detection through four fluorescence channels (Ch1-T1, ChS1-T2, ChS2-T3, and ChS3-T4). The placental images analyzed corresponded to a total of 21 fields of view (FOVs), digitized at 40× magnification with an individual surface area of 0.125491 mm^2^ per FOV. These fields were distributed across the experimental groups FA/FA, FA/NFA, NFA/NFA, and NFA/FA, with total analyzed areas of 0.627455 mm^2^, 0.752946 mm^2^, 0.627455 mm^2^, and 0.627455 mm^2^, respectively. Cell type identification was based on morphometric parameters previously described in the literature, particularly nuclear area, where cutoff points were established to distinguish the three cellular groups. This information enabled the construction of scatter plots correlating nuclear area with mean fluorescence intensity (area–mean intensity), facilitating the delineation of the different cell types through the application of regions of interest or “gates”. Finally, the defined cell types were visualized and differentiated using the nuclear marker in channel Ch1-T1, and the cutoff points used in the analysis were validated to ensure accuracy in cell classification. Once nuclear detection and separation of the cellular populations were completed, the area (µm^2^) and fluorescence intensity of the WGA, SVCT2, and GLUT1 markers were measured individually for each cell (Supplementary Document S2).

### 2.9. Spectral Confocal Microscopy

To determine whether the transporters are expressed at the plasma membrane level, WGA expression was colocalized with GLUT1 and SVCT2. Image processing and colocalization analysis were conducted using IMARIS v9.2 software (Bitplane, Oxford Instruments, Zurich, Switzerland). Manders’ overlap coefficient was used for graphical representation.

### 2.10. Statistical Analysis

All quantitative data were expressed as mean ± standard error of the mean (SEM). The distribution of each dataset was evaluated using the D’Agostino–Pearson omnibus normality test. Since most variables did not meet the assumption of normality, non-parametric tests were applied. Comparisons between the four experimental groups (FA/FA, FA/NFA, NFA/FA, and NFA/NFA) were performed using the Kruskal–Wallis test. When significant differences were detected, Dunn’s multiple comparison post hoc test was used to identify specific group differences with respect to the control (FA/FA). These analyses were applied to the following outcomes: (i) nuclear area; (ii) area and fluorescence intensity of GLUT1 and SVCT2 expression in cytotrophoblasts, syncytiotrophoblasts, and endothelial cells; and (iii) Manders’ overlap coefficients for transporter/membrane colocalization. A 95% confidence level was used, with statistical significance defined as *p* < 0.05. All analyses were conducted using GraphPad Prism version 10.0 for macOS (GraphPad Software, San Diego, CA, USA).

## 3. Results

### 3.1. Air Pollution Exposure

The daily average pollutant concentrations at the exposure site were 48.8 µg/m^3^ (±36.1; CV = 74%; CI_95%_ = 42–56) for PM2.5, 56.9 mg/m^3^ (±38.3; CV = 67.3%; CI_95%_ = 33–40) for PM10, and 0.78 ppm (±0.49; CV = 61.5%) for CO (Table 1). In the NFA chamber, PM2.5 averaged 44.6 µg/m^3^ (±9.8; CV = 21.9%; CI_95%_ = 0.7–0.88), closely matching ambient levels, whereas in the FA chamber, it was significantly reduced to 3.0 µg/m^3^ (±1.3; CV = 34.3%; *p* < 0.001), achieving a 94% reduction. Maternal exposure was estimated by multiplying the total inhaled air volume (1299 L over 22 days at 0.041 L/min) by the average PM2.5 concentration. NFA/NFA females inhaled 57,934 µg/m^3^ cumulatively (2633.40 µg/m^3^ daily), while FA/FA females were exposed to 3896.97 µg/m^3^ cumulatively (177.14 µg/m^3^ daily). The PM2.5 concentration curves during the study period are presented in Supplementary Document S3.

### 3.2. Nuclear Area and WGA Expression

**Figure 2:**
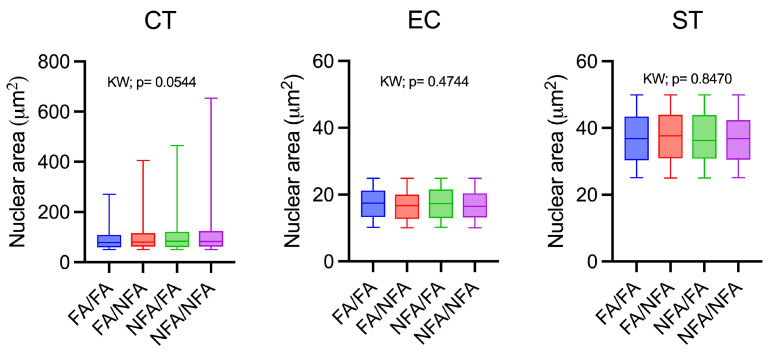
Analysis of the nuclear area of cytotrophoblasts (CTs), endothelial cells (ECs), and syncytiotrophoblasts (STs) in the labyrinth zone of the rat placenta at 21 days post-fertilization (dpf) following exposure to wood smoke-derived PM2.5 (n = 12 animals per group).

The cellular populations of cytotrophoblasts, syncytiotrophoblasts, and endothelial cells were accurately detected in the placental fields through digital processing with StrataQuest software (version 7.1.1.138; TissueGnostics GmbH, Vienna, Austria), using regions of interest or “gates” and applying previously described nuclear area (Supplementary Document S4). The immunofluorescence images, labeled with WGA to delineate the cells and DAPI for the nuclei, enabled the identification and classification of the cellular populations, while quantification of the nuclear area revealed no significant differences between experimental groups in cytotrophoblasts (*p* = 0.0544), endothelial cells (*p* = 0.4744), or syncytiotrophoblasts (*p* = 0.8470). This indicates that exposure to PM2.5 does not induce significant morphological changes in the nuclear area of these placental cells, despite its effects on transporter expression (Figure 2). Exposure to wood smoke-derived PM2.5 significantly reduced the expression area (μm^2^) and fluorescence intensity of the WGA marker in the placenta of Sprague–Dawley rats at 21 days of gestation compared to the control group (FA/FA; *p* < 0.0001), primarily in syncytiotrophoblasts and endothelial cells (Table 1).

### 3.3. Expression of GLUT1 and SVCT2 Transporters in the Placental Labyrinth Zone

**Figure 3:**
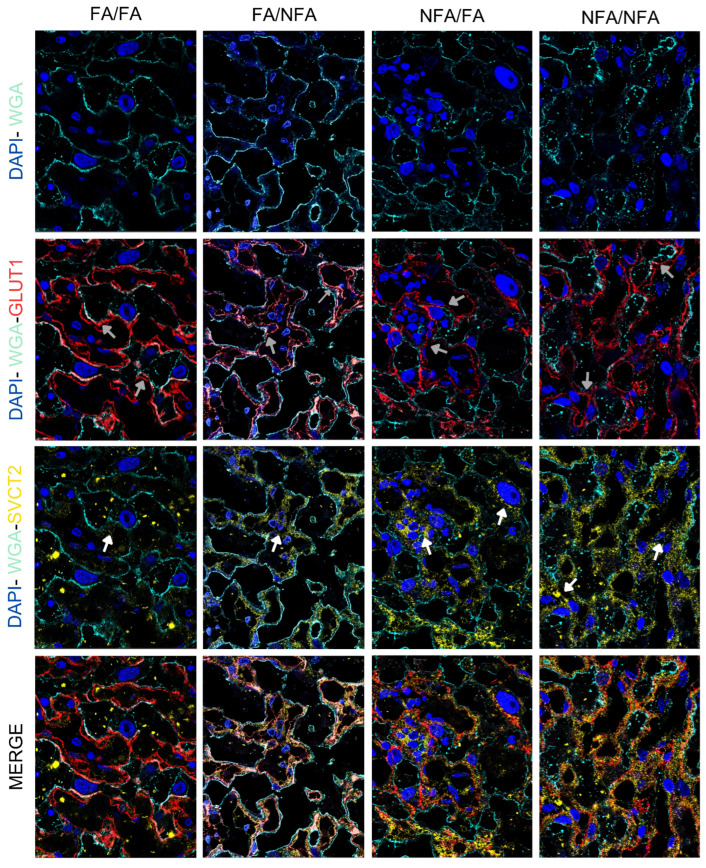
Representative confocal immunofluorescence images in placental labyrinth zone in 21-day post-fertilization (21-dpf) Sprague–Dawley rats (n = 12 animals per group). The panel shows DAPI (nuclei, blue), wheat germ agglutinin (WGA, cell membranes, light blue; white arrow), SVCT2 (yellow), and GLUT1 (red) staining, with the merged images demonstrating colocalization. Experimental groups are labeled as FA/FA, FA/NFA, NFA/FA, and NFA/NFA. In the FA/FA (control) group, intense and uniform colocalization between SVCT2 (yellow) and GLUT1 (red; gray arrow) was observed on the cell membranes delineated with WGA (light blue), along with well-defined nuclei labeled with DAPI (blue). The WGA marker clearly outlined the cell membranes, and the SVCT2 and GLUT1 signals appeared organized and overlapped across multiple membranous regions. In the FA/NFA group, SVCT2 and GLUT1 colocalization was less intense than in the FA/FA group. The WGA signal enabled the visualization of recognizable cell membranes, with regular morphology and defined contours. SVCT2 and GLUT1 distribution was visible, although with less overlap than in the control group. In the NFA/FA group, SVCT2 signal showed a more heterogeneous and more intense distribution compared to FA/FA, while GLUT1 exhibited localized expression. The WGA pattern remained defined, although regions with discontinuity in the cell contours were identified. In the NFA/NFA group, the signal intensity of SVCT2 and GLUT1 was increased, and their localization displayed a higher degree of heterogeneity. WGA staining was less intense and more fragmented, with a loss of clarity in cell boundary definition (magnification: 40×).

Regarding the expression area (µm^2^) of the GLUT1 and SVCT2 transporters, pregestational and/or gestational exposure to wood smoke-derived PM2.5 was associated with a lower mean of positive staining for GLUT1 compared to the control group (FA/FA; *p* < 0.0001). Exclusive gestational exposure to PM2.5 (FA/NFA) was associated with a lower mean area for SVCT2 compared to the control group (*p* < 0.0001). As for fluorescence intensity, exclusive gestational exposure to PM2.5 was associated with a lower mean intensity for GLUT1 and SVCT2 compared to the control group (*p* < 0.0001). However, pregestational exposure (NFA/FA and NFA/NFA) was associated with a higher mean fluorescence intensity for GLUT1 and SVCT2 compared to the control group ((*p* < 0.0001); Table 2). Figure 3 presents representative images obtained by confocal microscopy of rat placental tissue. In the FA/FA (control) group, there was intense and uniform colocalization of SVCT2 (yellow) and GLUT1 (red) along the cell membranes (WGA, light blue), with well-defined nuclei (DAPI, blue). WGA clearly delineated the cell membranes, showing close and organized colocalization with SVCT2 and GLUT1, indicating an orderly distribution of these transporters at the cell boundary. In the FA/NFA group, localization of SVCT2 and GLUT1 appeared less intense than in FA/FA. The membranes were recognizable with WGA, revealing a regular and uniform morphology, indicating that exposure to PM2.5 during gestation did not substantially affect cellular architecture. In contrast, the NFA/FA group showed a more heterogeneous distribution of SVCT2, with more intense staining compared to FA/FA, while GLUT1 maintained localized but less pronounced expression. The WGA signal remained relatively defined, although regions with discontinuity were evident. Finally, in the NFA/NFA group, an increase in intensity and an increase in the heterogeneity of SVCT2 and GLUT1 localization were observed. Partial internalization of GLUT1 into the cytoplasm, especially in the syncytiotrophoblast, was noted, along with the proximity of SVCT2 to perinuclear regions. WGA staining was less intense and more fragmented, with a loss of sharpness at the cell edges. This suggests that PM2.5 exposure affects the proximity between SVCT2 and WGA domains, possibly altering the cells’ ability to uptake vitamin C. GLUT1 was observed not only on the plasma membrane but also internalized into the cytoplasm, particularly in the syncytiotrophoblast, especially in the NFA/NFA group. SVCT2 expression was localized both on the plasma membrane and in regions near the cell nucleus in the most exposed groups, corresponding to the greater heterogeneity observed.

### 3.4. GLUT1 Transporter Expression by Cell Type

**Figure 4:**
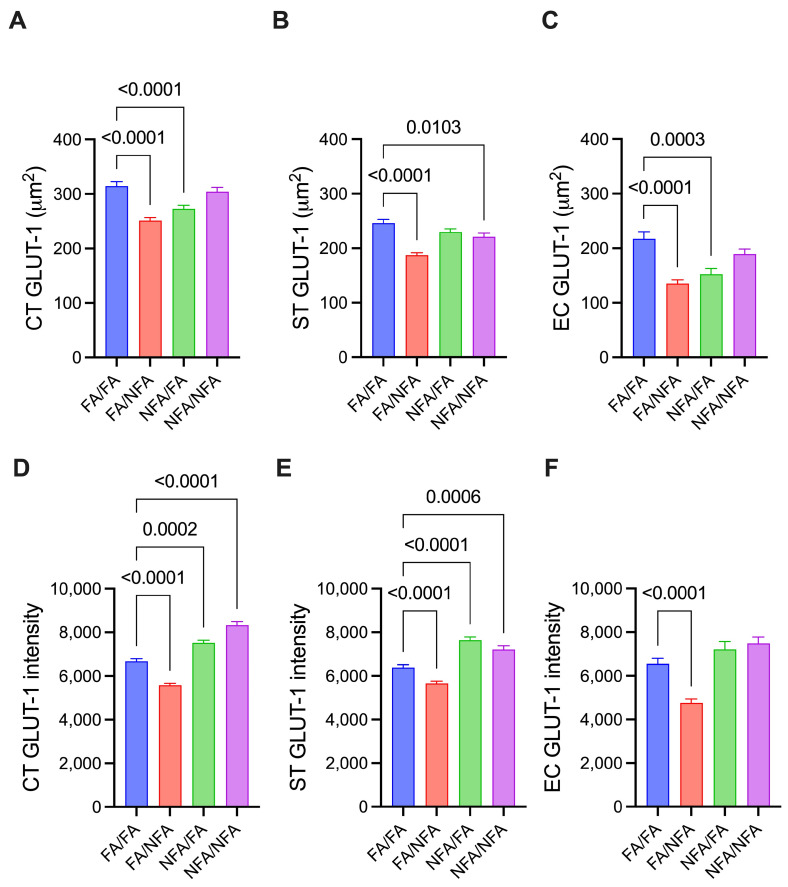
Expression of the GLUT1 transporter in different cell types of the placenta in 21-day post-fertilization (21-dpf) Sprague–Dawley rats (n = 12 animals per group; mean ± SEM). The expression is shown in three cell types: cytotrophoblast (CT), syncytiotrophoblast (ST), and endothelial cells (ECs). Panels (**A**–**C**) corresponding to the area (µm^2^) in CT, ST, and EC, respectively, and panels (**D**–**F**) corresponding to the fluorescence intensity in CT, ST, and EC, respectively, under different experimental conditions (Kruskal–Wallis; *p* < 0.05).

The detailed results of fluorescence area and intensity corresponding to the expression of GLUT1 and SVCT2 in the different analyzed cell types are provided in Supplementary Document S5. GLUT1 expression showed significant differences in placental cells depending on pregestational and/or gestational exposure to PM2.5. In cytotrophoblasts (Figure 4A), exclusive pregestational exposure (NFA/FA) decreased the GLUT1 area compared to the control (FA/FA; *p* < 0.0001), as did exclusive gestational exposure (FA/NFA; *p* < 0.0001). In syncytiotrophoblasts (Figure 4B), both cumulative chronic exposure (NFA/NFA; *p* = 0.0103) and exclusive gestational exposure (FA/NFA; *p* < 0.0001) resulted in a reduction compared to the control. In endothelial cells (Figure 4C), FA/FA showed a greater area than FA/NFA (*p* < 0.0001) and NFA/FA (*p* = 0.0003). These results indicate that pregestational and gestational exposure to PM2.5 differentially modulates GLUT1 transporter expression in placental cells, with a specific response pattern depending on the cell type. This suggests differential regulation of GLUT1 that could affect glucose availability and, consequently, the metabolic environment during placental development. The intensity of GLUT1 expression in placental cells also varied significantly depending on pregestational and gestational exposure to PM2.5. In cytotrophoblasts (Figure 4D) and syncytiotrophoblasts (Figure 4E), a similar pattern was observed: cumulative chronic exposure (NFA/NFA) and exclusive pregestational exposure (NFA/FA) significantly increased the intensity of GLUT1 expression compared to the control group (FA/FA). In contrast, exclusive gestational exposure (FA/NFA) significantly reduced the intensity of GLUT1 expression in both cell types compared to the control (*p* < 0.0001). In endothelial cells (Figure 4F), exclusive gestational exposure (FA/NFA) significantly decreased GLUT1 intensity compared to the control (*p* < 0.0001). These results indicate that the intensity of GLUT1 expression in placental cells is strongly influenced by the timing of PM2.5 exposure, showing a reduction during exclusive gestational exposure and an increase following pregestational and cumulative chronic exposures. Moreover, the differential response observed among the various cell types suggests specific vulnerability of cytotrophoblasts, syncytiotrophoblasts, and endothelial cells to environmental conditions, which could impact glucose transport during placental development.

### 3.5. SVCT2 Transporter Expression by Cell Type

**Figure 5:**
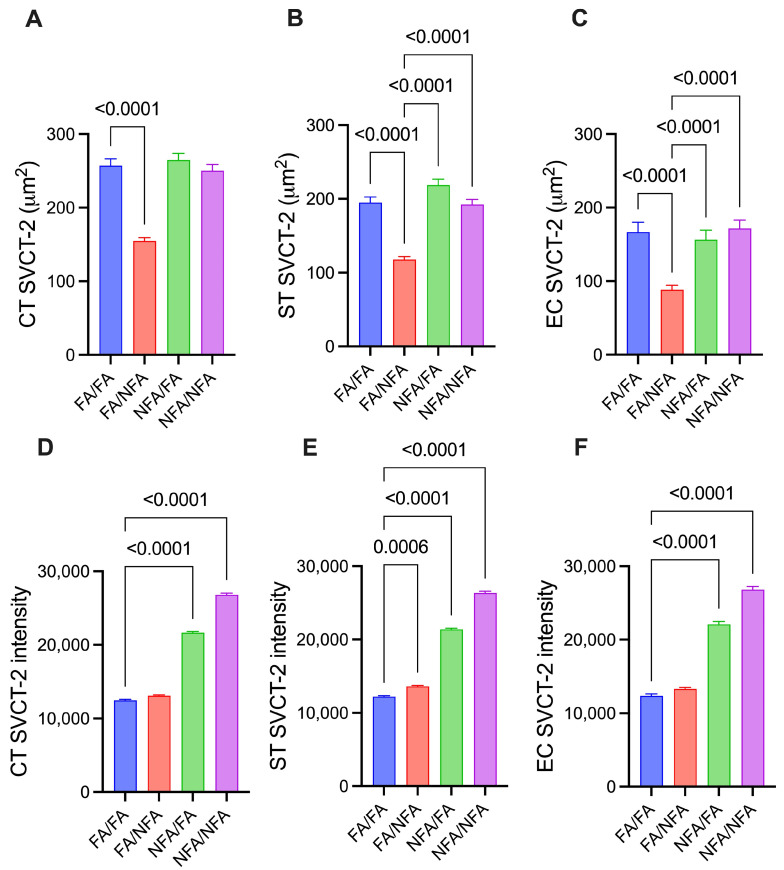
Expression of the SVCT2 transporter in different cell types of the placenta in 21-day post-fertilization (21-dpf) Sprague–Dawley rats (n = 12 animals per group; mean ± SEM). The expression is shown in three cell types: cytotrophoblast (CT), syncytiotrophoblast (ST), and endothelial cells (ECs). Panels (**A**–**C**) corresponding to the area (µm^2^) in CT, ST, and EC, respectively, and panels (**D**–**F**) corresponding to the fluorescence intensity in CT, ST, and EC, respectively, under different experimental conditions (Kruskal–Wallis; *p* < 0.05).

The expression area of SVCT2 in the group exposed exclusively during gestation (FA/NFA) showed a significant reduction in both cytotrophoblasts (Figure 5A; *p* < 0.0001) and syncytiotrophoblasts (Figure 5B; *p* < 0.0001) compared to the control group. Additionally, pregestational exposure (NFA/FA and NFA/NNA) was associated with an increase relative to the control group (FA/FA). In endothelial cells (Figure 5C), exclusive gestational exposure (FA/NFA) significantly reduced the SVCT2 area compared to the control (FA/FA; *p* < 0.0001). SVCT2 transporter expression varied significantly according to cell type and exposure conditions. Gestational exposure to PM2.5 (FA/NFA) reduced the SVCT2 area, while pregestational (NFA/FA) and cumulative chronic exposure (NFA/NNA) tended to restore or increase its expression, suggesting a differential, cell type-specific response to environmental exposure. The intensity of SVCT2 expression also differed depending on the period of exposure to wood smoke-derived PM2.5 across the placental cell types evaluated. In cytotrophoblasts (Figure 5D), exclusive pregestational (NFA/FA) and cumulative chronic (NFA/NNA) exposure significantly increased SVCT2 intensity compared to the control (FA/FA; *p* < 0.0001). In syncytiotrophoblasts (Figure 5E), both pregestational and/or gestational exposure significantly increased SVCT2 intensity compared to the control. In endothelial cells (Figure 5F), both pregestational (NFA/FA) and chronic (NFA/NFA) exposure significantly increased intensity relative to the control (*p* < 0.0001). These results demonstrate that the intensity of SVCT2 transporter expression in placental cells is strongly influenced by the period of exposure to PM2.5, showing greater vulnerability during pregestational exposure. In contrast, gestational exposure tends to increase SVCT2 intensity, suggesting a differential response depending on cell type and timing of exposure, with potential implications for vitamin C transport capacity during placental development.

### 3.6. Distribution and Colocalization of GLUT1 and SVCT2 Transporters

**Figure 6:**
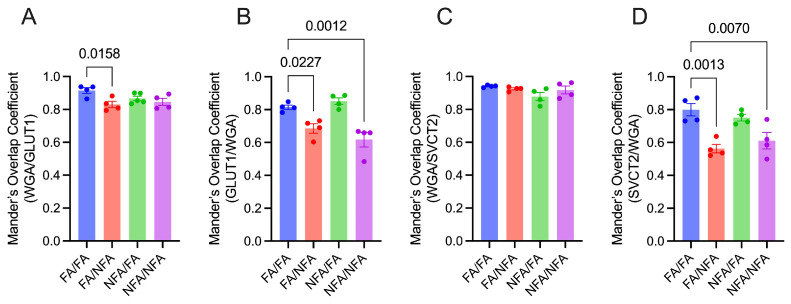
Cellular distribution of GLUT1 and SVCT2 transporters in the placenta of 21-day post-fertilization (21-dpf) Sprague–Dawley rats (n = 12 animals per group) evaluated using Manders’ overlap coefficients (mean ± SEM). To determine the relative localization of the transporters at the plasma membrane, wheat germ agglutinin (WGA) was used as a plasma membrane marker, and its colocalization with GLUT1 and SVCT2 was analyzed under different experimental conditions. Manders’ overlap coefficient was calculated for the colocalization of (**A**) WGA and GLUT1, (**B**) GLUT1 and WGA, (**C**) WGA and SVCT2, and (**D**) SVCT2 and WGA, revealing significant differences in colocalization across experimental groups. Values above the bars indicate statistically significant differences (*p* < 0.05) between groups as determined by statistical analysis. These findings suggest differential regulation of the subcellular localization of the transporters under the evaluated experimental conditions.

**Figure 7:**
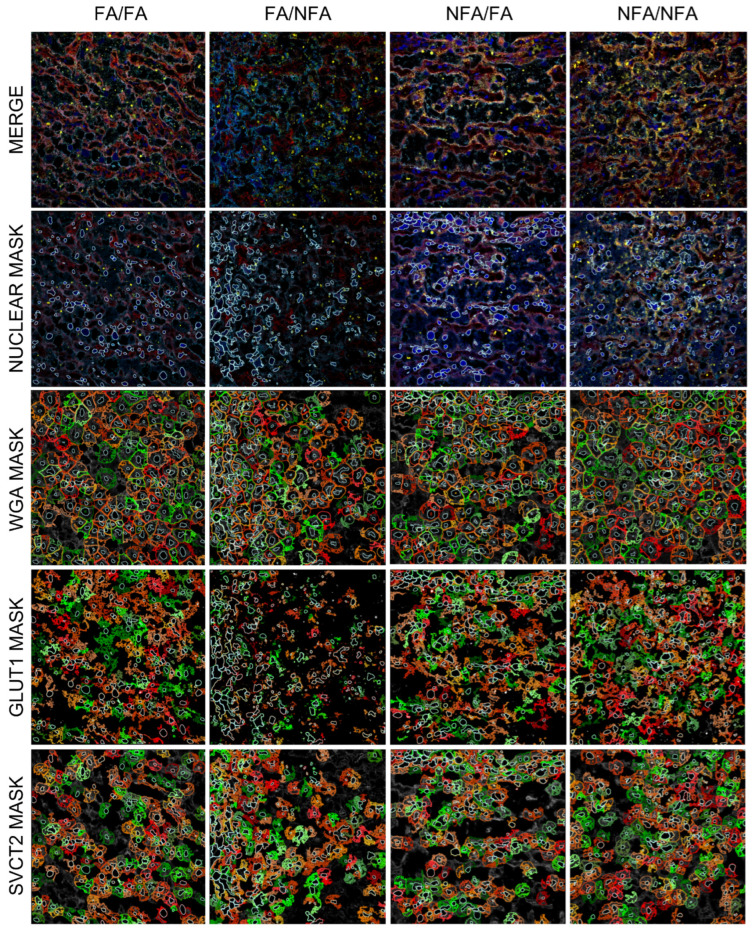
Subcellular localization of WGA and the transporters SVCT2 and GLUT1 in the placenta of pregnant rats (n = 12 animals per group) at 21 days post-fertilization (dpf), analyzed using masks generated with StrataQuest 7.1.1.138 software to delineate the subcellular expression of the markers. A total of 21 fields of view (FOVs) per experimental group (FA/FA, FA/NFA, NFA/FA, NFA/NFA), each digitized at 40× magnification with an individual surface area of 0.125491 mm^2^, were analyzed using the Cell Analysis and Classification module. Exclusive gestational exposure was associated with reduced colocalization of GLUT1 with WGA, showing decreased expression and predominantly cytoplasmic localization. In contrast, pregestational exposure was associated with SVCT2 internalization, with expression observed both on the plasma membrane and in the cytoplasm. Mask details: nuclear mask delineates the cell nucleus; WGA mask marks the plasma membrane; GLUT1–SVCT2 mask shows the subcellular distribution of the transporters. Colors in the masks vary to distinguish cells and are randomly assigned by the software (magnification: 40×).

When assessing the colocalization of both transporters relative to WGA, a reduction was observed, particularly in the groups exposed to PM2.5 during gestation. Statistical analysis of the Manders’ colocalization coefficients for the combinations WGA/GLUT1, GLUT1/WGA, WGA/SVCT2, and SVCT2/WGA across the four groups indicated significant differences in the colocalization of these molecules, likely influenced by exposure to filtered air (FA) or unfiltered air (NFA). For WGA/GLUT1, the colocalization coefficient in the FA/FA group was significantly higher than in the FA/NFA group (*p* = 0.0158; Figure 6A). This suggests that exposure to unfiltered air during gestation (FA/NFA) affects the interaction or spatial proximity between WGA and GLUT1, potentially altering GLUT1-mediated glucose uptake processes in the cells. Regarding GLUT1/WGA, the coefficient showed a significant reduction in the FA/NFA and NFA/NNA groups compared to FA/FA (*p* = 0.0012 and *p* = 0.0227, respectively; Figure 6B). In contrast to the FA/FA group, where GLUT1 expression was abundant at the cellular level and distributed both within the cytoplasm and on the plasma membrane colocalizing with WGA, exposure to PM2.5 disrupted this pattern. For WGA/SVCT2, although no specific significant differences were detected between groups, values tended to be higher and more homogeneous in the FA/FA group, indicating that exposure to filtered air favors interaction between WGA and SVCT2, a key protein in vitamin C transport (Figure 6C). Finally, for SVCT2/WGA, the FA/NFA and NFA/NFA groups showed significantly lower expression values compared to FA/FA (*p* = 0.0013 and *p* = 0.0070, respectively; Figure 6D). This observed pattern reinforces the hypothesis that exposure to PM2.5 during the pregestational or gestational period negatively alters the distribution of GLUT1 in relation to WGA-labeled domains. As shown in Figure 7, GLUT1 transporter expression was markedly reduced in the groups exposed during gestation and was predominantly observed in the cytoplasm near the cell nucleus, rather than on the plasma membrane where WGA is expressed. Consistently, SVCT2 distribution was predominantly membrane-associated across all experimental groups; however, in the groups exposed during the gestational period, greater cytoplasmic localization of the transporter was evident, indicating a more internalized expression pattern (Figure 7).

## 4. Discussion

The findings of this study contribute to understanding the impact of fine particulate matter (PM2.5) derived from wood smoke on placental function, particularly regarding the regulation of glucose and vitamin C transport. It is noteworthy that in a previous study conducted by our group, we demonstrated that exposure to PM2.5 induces placental hypoxia and reduces both fetal weight and length [1,30], thereby complementing the morphological observations and transporter redistribution in the placenta described in the present study. Taken together, the results obtained through confocal microscopy in the placental labyrinth zone of rats revealed differences in the localization and organization of SVCT2 and GLUT1 transporters depending on pregestational and gestational exposure history to PM2.5. The more intense expression and organized colocalization of both transporters in the control group (FA/FA) contrasted with the diminished and dispersed signal observed in the NFA/NFA group, which was subjected to continuous exposure to an unfiltered environment. The intermediate groups (FA/NFA and NFA/FA) exhibited variable patterns, suggesting that both pregestational and gestational exposure differentially influenced placental expression of these transporters. The alterations observed in the WGA signal, particularly in the FA/NFA group, reflected a loss of integrity in the cell membranes. These findings suggest that gestational exposure to PM2.5 compromises not only the expression of essential transporters for maternal–fetal exchange but also the structural organization of the placenta, which could have functional implications for nutrient transport efficiency during embryonic development. Biologically, these findings demonstrate that PM2.5, especially when present during both the pregestational and gestational stages, compromises the morphological integrity of placental tissue, potentially affecting its barrier and maternal–fetal exchange functions. In this context, the exclusive use of quantitative immunofluorescence with confocal microscopy was selected to preserve tissue architecture and enable precise discrimination of cell type-specific alterations in transporter expression and subcellular localization. This approach, supported by a standardized digital analysis protocol, allowed the simultaneous quantification of staining area, fluorescence intensity, and colocalization with membrane markers—parameters that cannot be resolved by techniques such as Western blot or qPCR, which require tissue homogenization and result in loss of spatial information. While complementary analyses of protein or mRNA levels could further substantiate these findings, the methodological focus on spatial distribution provided critical insights into the compartment-specific effects of PM2.5 exposure within the placental labyrinth. To ensure the reproducibility and robustness of the quantitative immunofluorescence data, four non-overlapping fields of view (FOVs) per placental section were analyzed for each animal, with consistent results obtained across biological replicates within each experimental group. This consistency across both technical and biological replicates supports the reliability of the fluorescence measurements and strengthens the confidence in the quantitative accuracy of our analysis.

Pregestational and gestational exposure to wood smoke-derived PM2.5 does not affect the nuclear area of the cells that constitute the placental labyrinth (cytotrophoblasts, syncytiotrophoblasts, and endothelial cells); however, exclusive gestational exposure significantly reduces both the area and fluorescence intensity of GLUT1 and SVCT2 in these cells. This reduction may compromise the placenta’s capacity to protect against oxidative damage and decrease the efficiency of glucose transport, thereby affecting the energy supply to the developing fetus. The absence of changes in the nuclear area of labyrinthine placental cells following gestational or pregestational exposure to PM2.5 suggests that nuclear morphology remains preserved despite functional alterations, evidenced by the decrease in the area and fluorescence intensity of GLUT1 and SVCT2 transporters. This finding indicates that PM2.5-induced damage selectively affects cellular compartments such as the cytoplasm and plasma membrane, without compromising nuclear architecture, which could reflect adaptive mechanisms or a differential sensitivity of cellular compartments to environmental exposure. Consequently, these cellular alterations are associated with smaller fetuses at the end of gestation [1,30]. In contrast, pregestational exposure was associated with an increase in SVCT2 expression area as well as higher fluorescence intensity for both transporters. The increase in SVCT2 expression observed following pregestational exposure may be related to a compensatory mechanism aimed at counteracting oxidative stress induced by chronic hypoxia, given the antioxidant role of vitamin C. This elevation in transporter levels suggests an adaptive placental response intended to preserve fetal integrity against oxidative damage [13]. Some studies have suggested that the adverse effects caused by prolonged exposure to atmospheric pollutants such as PM2.5, PM10, NO2, and NOx can be mitigated through the consumption of antioxidants [21,38]. Vitamin C can attenuate oxidative damage, inflammation, and hypoxia-induced apoptosis [39]. At the subcellular level, we observed that SVCT2 is internalized in trophoblasts in groups exposed gestationally. In these cases, the protein displays a dual localization, being distributed both on the plasma membrane and in perinuclear regions of the trophoblastic tissue. This redistribution, induced by gestational exposure to PM2.5, may represent a cellular protective mechanism aimed at preserving critical structures such as the nucleus from oxidative damage caused by exposure. It has been demonstrated that in trophoblastic cells, supplementation with vitamins C and E can reduce mitochondrial damage, thereby enhancing cell survival [40]. However, this change could compromise the efficiency of vitamin C transport across the plasma membrane, limiting its availability within the intracellular environment to counteract oxidative stress. Regarding GLUT1, it has been observed that hypoxia upregulates HIF-1α and GLUT1, increasing the density of this transporter to optimize glucose transport [41]. This mechanism is likely activated only during pregestational exposure, but not during gestational exposure to PM2.5, where a reduction in GLUT1 was observed. Similar responses have been reported in cases of preeclampsia and intrauterine growth restriction (IUGR), where severe placental hypoxia reduces GLUT1 expression [42]. In terms of cell type, when a reduction in GLUT1 intensity occurs, it is more pronounced in cytotrophoblasts (CTs) and syncytiotrophoblasts (STs). Previous studies suggest that these cells are particularly sensitive to hypoxia and oxidative stress, which impairs their ability to express these transporters [43,44,45]. In the syncytiotrophoblast, GLUT1 densities can be regulated independently on the maternal and fetal surfaces, with substantially higher abundance in the maternal-facing microvilli than in the basal membrane [41,46]. The decrease in GLUT1 in both layers may suggest that the maternal side of the placenta is more affected by gestational exposure to PM2.5, a hypothesis that requires confirmation in future studies. The colocalization of GLUT1 with WGA in the control group demonstrated that the transporters were predominantly located on the plasma membrane of placental cells. However, in the PM2.5-exposed groups, an increase in cytoplasmic GLUT1 was observed, particularly evident in the syncytiotrophoblast, which could reflect a dysfunctional mechanism of subcellular transport induced primarily by gestational exposure to PM2.5. Given the essential role of GLUT1 in glucose transport, a redistribution to intracellular compartments may reduce glucose availability in the extracellular space, affecting the fetus’s energy metabolism, as GLUT1 expression in the basal plasma membrane has also been positively correlated with fetal birth weight [47].

**Figure 8:**
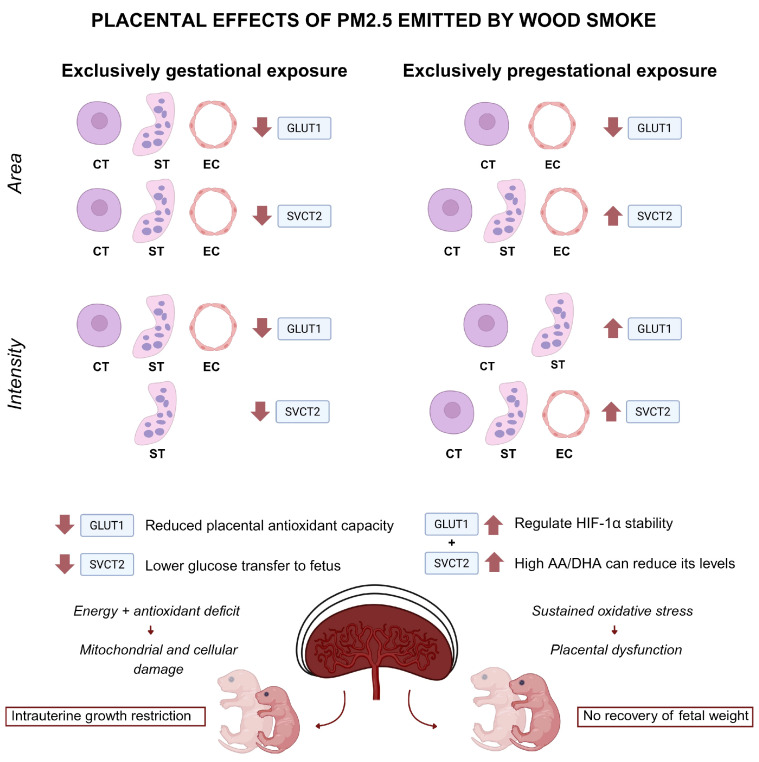
Placental effects of wood smoke-derived PM2.5 exposure on GLUT1 and SVCT2 expression in the labyrinth zone of rat placenta (n = 12 animals per group). Quantitative immunofluorescence analysis revealed differential effects of exclusive gestational versus exclusive pregestational exposure to PM2.5 on the expression area and fluorescence intensity of the glucose transporter GLUT1 and the sodium-dependent vitamin C transporter SVCT2 in cytotrophoblasts (CTs), syncytiotrophoblasts (STs), and endothelial cells (ECs). Gestational exposure was associated with reduced membrane expression and intensity of both transporters, suggesting impaired glucose transfer to the fetus and diminished placental antioxidant capacity, leading to a combined energy and redox deficit, mitochondrial and cellular damage, and increased risk of intrauterine growth restriction (IUGR). In contrast, pregestational exposure induced a compensatory increase in SVCT2 and GLUT1 expression, potentially as an adaptive response to oxidative stress, contributing to the regulation of HIF-1α stability; however, this did not result in recovery of fetal weight and was associated with persistent oxidative stress and placental dysfunction.

Under normal conditions, vitamin C levels decrease near the end of gestation, possibly due to higher requirements at this stage [13]. The increased expression of SVCT2 observed in groups exposed to PM2.5 during the pregestational period may represent a compensatory response aimed at mitigating hypoxia-induced oxidative stress by facilitating the uptake of AA into the cell, a process shown to have protective effects [38]. However, the capacity of this compensatory response to counteract the adverse effects of prolonged PM2.5 exposure is limited, as sustained oxidative stress may overwhelm the placenta’s antioxidant defenses, exacerbating cellular damage and contributing to placental dysfunction. Additionally, it has been reported that the joint activity of SVCT2 and GLUT1 plays an important role in regulating the stability of HIF-1α, as elevated levels of AA and its derivative dehydroascorbic acid (DHA) can reduce the levels of this factor [18]. Nevertheless, the potential compensatory effect expected from increased AA uptake was not reflected in fetal weight recovery. Therefore, it can be hypothesized that increased AA transport in the placenta could lead to cellular saturation, exacerbating oxidative stress, causing extensive DNA damage, and depleting ATP reserves, ultimately resulting in metabolic exhaustion and a pro-oxidant effect [48]. In the case of exclusive gestational exposure, the reduction in GLUT1 area and intensity suggests a diminished capacity of the syncytiotrophoblast and cytotrophoblast to transport glucose, while decreased membrane expression of SVCT2 would limit AA uptake, thereby reducing antioxidant defense. This combination generates a dual deficit in energy substrates and redox balance, creating a suboptimal intrauterine environment that, in this model, is associated with reduced fetal growth and, clinically, with an increased risk of intrauterine growth restriction, placental insufficiency, and greater fetal susceptibility to oxidative injury, potentially leading to metabolic and cardiovascular disorders later in life (Figure 8).

The differential effects of PM2.5 exposure depending on the timing of exposure highlight both the adaptability and vulnerability of the placenta. Based on our findings, we predict that gestational or combined (pregestational and gestational) exposure to wood smoke-derived PM2.5, by reducing the expression and altering the subcellular distribution of GLUT1 and SVCT2, will result in decreased fetal weight and an increased risk of gestational complications, such as intrauterine growth restriction. In contrast, exclusive pregestational exposure, by inducing a compensatory increase in the expression of these transporters, may partially mitigate the impact of oxidative stress, leading to a lesser degree of fetal growth impairment compared to groups exposed during gestation. These results underscore the need for future research to evaluate the impact of antioxidant interventions in human models exposed to PM2.5.

Limitations. Although Sprague–Dawley rats are an established model for reproductive toxicology [49], the hemotrichorial placenta of rats differs from the human hemomonochorial or dichorial placenta, which may influence sensitivity to PM2.5, the regulation of transporters such as GLUT1 and SVCT2, and the extrapolation of results to humans [50]; future studies in models more closely related to humans or clinical trials could address this limitation. The evaluation of GLUT1 and SVCT2 was limited to immunofluorescence due to resource constraints, without complementary Western blot or qPCR analyses to confirm protein or transcriptomic changes, although a standardized digital quantification protocol partially mitigated this limitation; additional techniques and antibody validation would be necessary in future research. The absence of hypoxia biomarkers (e.g., HIF-1α) or oxidative stress markers (e.g., MDA) in this study, although previously evaluated [1], limits direct correlation with transporter changes, suggesting the inclusion of these markers in subsequent investigations. Analysis at a single gestational time point (day 21) prevented exploration of the temporal progression of placental damage, although the differentiated exposure groups (pregestational and gestational) allowed discrimination of period-specific effects; a temporal analysis at multiple gestational stages would enrich the findings. The exclusive focus on GLUT1 and SVCT2 excluded other transporters (e.g., GLUT3, amino acid transporters), and the lack of data on maternal health (e.g., systemic inflammation) restricts a comprehensive understanding of the impact of PM2.5. Although average PM2.5 concentrations in the FA (3.0 µg/m^3^) and NFA (44.6 µg/m^3^) chambers were reported, with daily measurements included in supplementary documents and PM2.5 components previously assessed [30], future studies could analyze how specific variations in these components modulate placental effects. Long-term effects in offspring (G3) were not evaluated, representing an opportunity to investigate transgenerational impacts. Looking ahead, trials with antioxidant supplements during pregnancy, such as vitamins C and E, which have prevented preterm birth [51], could explore placental markers and fetal outcomes in women exposed to PM2.5, complementing these findings with studies evaluating additional transporters, maternal health, and the temporal progression of placental damage.

## 5. Conclusions

In conclusion, the alterations in GLUT1 and SVCT2 expression following pregestational and/or gestational exposure observed in this study demonstrate the susceptibility of the placenta to environmental pollutants generated by wood combustion for indoor heating. These changes, possibly mediated by oxidative stress and hypoxia [22], provide insight into how PM2.5 affects maternal–fetal exchange. The differential response of GLUT1 and SVCT2 according to the timing of PM2.5 exposure reveals fundamental aspects of placental adaptability but also highlights its vulnerability in sustaining fetal development under environmental stress. The findings of this study have significant implications for both obstetric clinical practice and public health. The alteration in the expression and localization of GLUT1 and SVCT2 in the placenta, induced by exposure to wood smoke-derived PM2.5, suggests that environmental pollution may compromise the supply of glucose and vitamin C to the fetus, affecting its intrauterine development. As the placenta is the primary organ for the exchange of nutrients and antioxidants between mother and fetus, disruption of these transporters could increase the risk of fetal growth restriction, placental oxidative stress, and gestational complications such as preeclampsia and preterm birth. From a public health perspective, these results reinforce the need to implement strategies to mitigate maternal exposure to air pollutants, particularly in populations highly dependent on biomass combustion for heating and cooking. Regulating PM2.5 emissions, alongside awareness campaigns on the risks associated with air pollution during pregnancy, could contribute to improved perinatal outcomes and reduce the burden of diseases associated with prenatal exposure to environmental pollutants.

## Figures and Tables

**Table 1:** Area and fluorescence intensity for WGA and DAPI in the placental labyrinth zone (mean ± SEM) in 21-day post-fertilization (21-dpf) Sprague–Dawley rats (n = 12 animals per group).

		FA/FA	FA/NFA	NFA/FA	NFA/NFA	*p*-Value
Area (um^2^)	WGA	521.2 ± 242.3 ^abc^	377.8 ± 221.1 ^a^	457.5 ± 266.9 ^b^	434.3 ± 248.6 ^c^	<0.0001
	DAPI	53.75 ± 39.88 ^a^	59.91 ± 48.17	63.97 ± 51.45 ^a^	59.09 ± 48.48	0.0016
Intensity	WGA	10,455 ± 2606 ^ab^	8562 ± 2304 ^a^	9948 ± 2660 ^b^	10,245 ± 3081	<0.0001

Values are mean ± SEM. Same letters indicate significant differences between groups (one-way ANOVA, Tukey’s test, *p* < 0.05). Groups sharing a letter do not differ significantly.

**Table 2:** Area and fluorescence intensity for GLUT1 and SVCT2 in the placental labyrinth zone (mean ± SEM) in 21-day post-fertilization (21-dpf) Sprague–Dawley rats (n = 12 animals per group).

		FA/FA	FA/NFA	NFA/FA	NFA/NFA	*p*-Value
Area (um^2^)	GLUT1	269 ± 138 ^abc^	209 ± 129 ^a^	242 ± 136 ^b^	252 ± 155 ^c^	<0.0001
	SVCT2	216 ± 151 ^a^	130 ± 112 ^a^	234 ± 178	214 ± 160.4	<0.0001
Intensity	GLUT1	6519 ± 2278 ^abc^	5493 ± 2407 ^a^	7532 ± 2860 ^b^	7738 ± 3542 ^c^	<0.0001
	SVCT2	12,352 ± 2284 ^abc^	13,345 ± 2835 ^a^	21,572 ± 3144 ^b^	26,621 ± 4871 ^c^	<0.0001

Values are mean ± SEM. Same letters indicate significant differences between groups (one-way ANOVA, Tukey’s test, *p* < 0.05). Groups sharing a letter do not differ significantly.

## Data Availability

The data presented in this study are openly available in Zenodo at https://doi.org/10.5281/zenodo.15809265 (accessed on 16 August 2025).

## Supplementary Materials

The following supporting information can be downloaded at: https://www.mdpi.com/article/10.3390/antiox14091050/s1, Figure S1: Exposure chambers in the courtyard of the Faculty of Medicine at the University de La Frontera, located in the downtown area of Temuco, 500 m from the environmental air monitoring station (−38.7496844990132, −72.6188400896599). Figure S2: Workflow of cell analysis and classification using StrataQuest software, illustrating representative steps of image processing to quantify GLUT1 and SVCT2 expression in distinct cellular populations. Figure S3: Daily PM2.5 concentrations (µg/m^3^) recorded at the Las Encinas Monitoring Station (Temuco, Chile) during the study period, differentiating validated, preliminary, and unvalidated records. Figure S4: Nuclear area analysis in placental tissue from pregnant rats exposed to PM2.5, with classification of cytotrophoblasts, syncytiotrophoblasts, and endothelial cells based on morphometric criteria across the four experimental groups. Table S1: Averages of particulate matter (PM) and CO recordings during the study period (15 June–30 September 2021) and annually. Table S2: Area and intensity of GLUT1 and SVCT2 in cytotrophoblasts, syncytiotrophoblasts, and endothelial cells.

## Abbreviations

The following abbreviations are used in this manuscript:

PM2.5	Particulate Matter 2.5
GLUT1	Glucose Transporter 1
SVCT2	Sodium-Dependent Vitamin C Transporter 2
FA	Filtered Air
NFA	Non-Filtered Air
ROS	Reactive Oxygen Species
OS	Oxidative Stress
RNS	Reactive Nitrogen Species
ATP	Adenosine Triphosphate
AA	Ascorbic Acid
DHA	Dehydroascorbic Acid
WHO	World Health Organization
HIF-1α	Hypoxia-Inducible Factor 1-alpha
CT	Cytotrophoblast
ST	Syncytiotrophoblast
ECs	Endothelial Cells
WGA	Wheat Germ Agglutinin
DAPI	4′,6-Diamidino-2-Phenylindole
FOV	Field of View
MERV8	Minimum Efficiency Reporting Value 8
HEPA	High-Efficiency Particulate Air
CO	Carbon Monoxide
NO2	Nitrogen Dioxide
dpf	Days Post-Fertilization
G1	First Generation
G2	Second Generation
G3	Third Generation
IUGR	Intrauterine Growth Restriction
MDA	Malondialdehyde
