# Supplementary material for: Impact of PM2.5 Emitted by Wood Smoke on the Expression of Glucose Transporter 1 (GLUT1) and Sodium-Dependent Vitamin C Transporter 2 (SVCT2) in the Rat Placenta: A Pregestational and Gestational Exposure Study
Source: Antioxidants (Basel). 2025 Aug 26;14(9):1050. doi: 10.3390/antiox14091050 (PMC12466713; doi:10.3390/antiox14091050)
Supplement: Supplementary file 1 [file antioxidants-14-01050-s001.zip › antioxidants-3768107-supplementary.pdf]

**Supplementary Document S1**

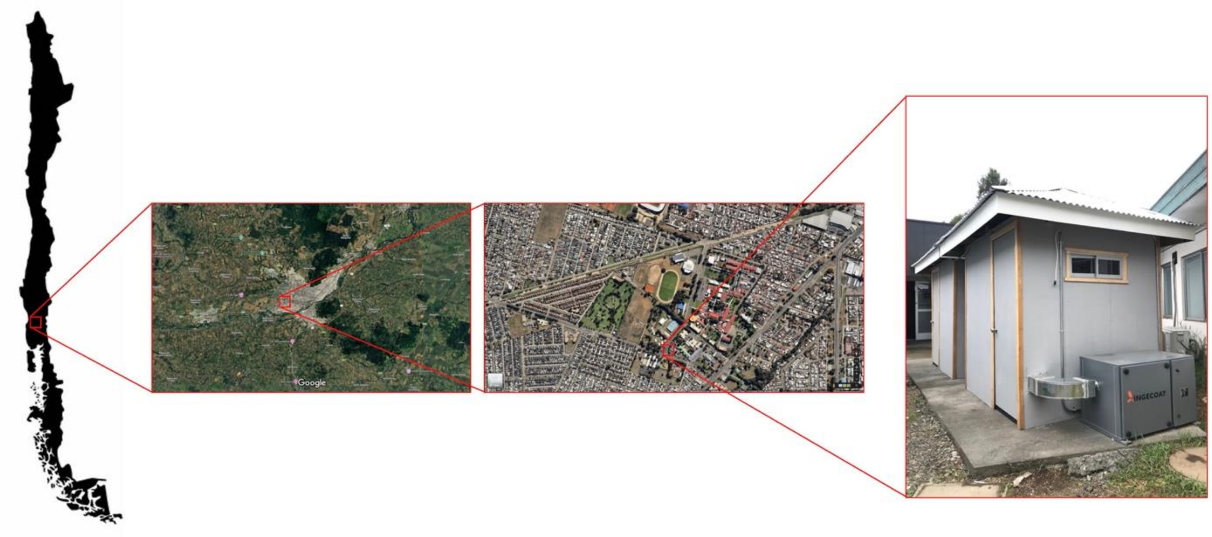


*Figure S1. Exposure chambers in the courtyard of the Faculty of Medicine at the University de La Frontera, located in the downtown area of Temuco, 500 meters away from the environmental air monitoring station (-38.7496844990132, -72.6188400896599).*

## Supplementary Document S2

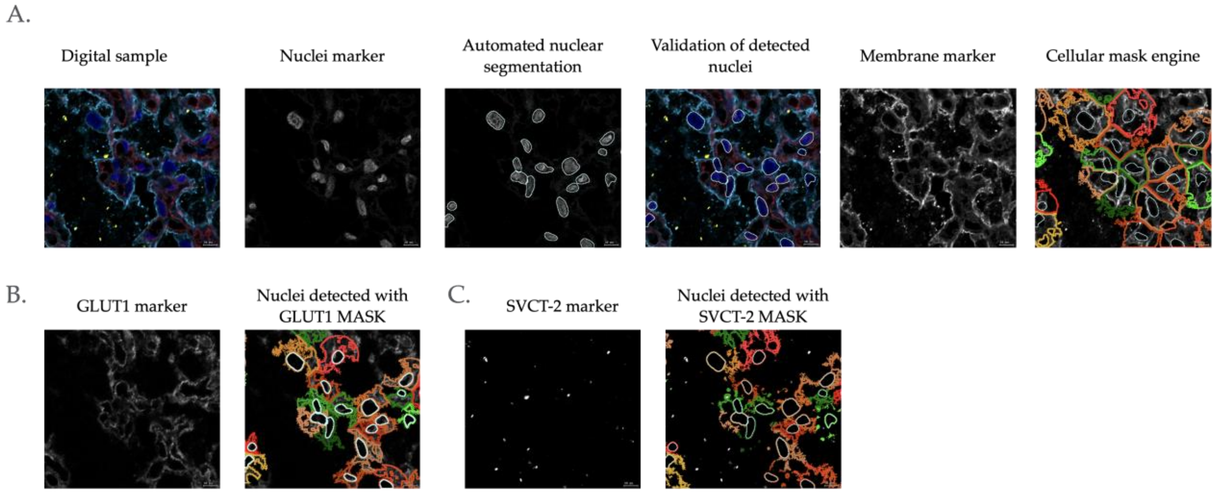


StrataQuest workflow for GLUT1 and SVCT-2 in placenta sections.
Scale bar 10 µm, ×40.
A. Nuclear and membrane segmentation.
B. Result of the GLUT1 transporter cellular mask engine.
C. Result of the SVCT-2 transporter cellular mask engine

*Figure S2. Workflow of cell analysis and classification using StrataQuest software. Representative steps of the image processing pipeline applied to placental sections to quantify the expression of GLUT1 and SVCT2 in distinct cellular populations. High-resolution confocal images (.czi files) were acquired with a Zeiss LSM 780 microscope and analyzed in StrataQuest v7.1.1.138. (A) Sequential workflow showing nuclear and membrane segmentation: digital sample (40× magnification), nuclear marker staining, automated nuclear segmentation, validation of detected nuclei, membrane marker staining, and final cell segmentation using the cellular mask engine. (B) Masking and segmentation results for GLUT1, illustrating the identification of nuclei within cellular contours defined by the GLUT1 marker. (C) Masking and segmentation results for SVCT2, showing nuclei within contours defined by the SVCT2 marker. Cytotrophoblasts (CT), syncytiotrophoblasts (ST), and endothelial cells (EC) were classified based on morphometric criteria, particularly nuclear area, as validated by scatter plot analysis correlating nuclear size and marker intensity. The analysis encompassed 21 fields of view (FOVs) per experimental group (FA/FA, FA/NFA, NFA/FA, NFA/NFA), with automated quantification of fluorescence area and intensity for each individual cell.*

## Supplementary Document S3

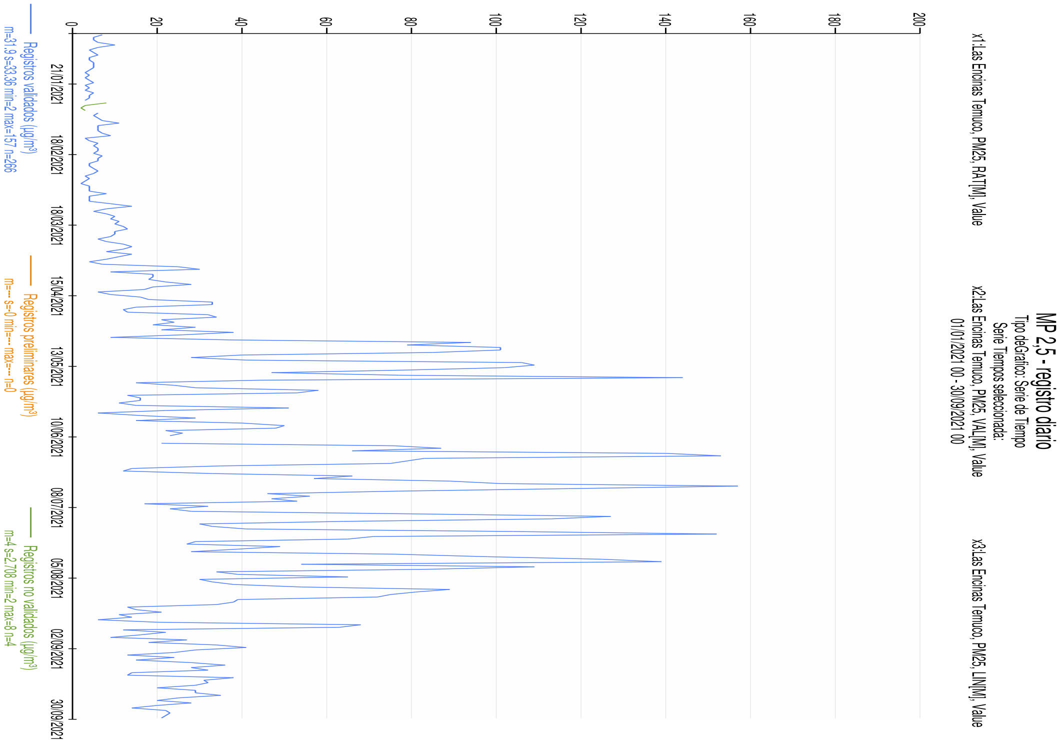


*Figure S3. Figure III. Daily PM2.5 concentrations (µg/m³) recorded at the "Las Encinas Monitoring Station" (Temuco, Chile) during the study period (January 1 to September 30, 2021). PM2.5 levels were measured externally using a beta attenuation monitor BAM 1020 (Met One Instruments, Inc., Grant Pass, OR, USA), equipped with a carbon-14 (60 µCi ±15 µCi) beta source and a photomultiplier tube beta detector with an organic plastic scintillator, operating at a flow rate of 16.7 L/min. Data were provided by "Algoritmos y Mediciones Ambientales SpA" and accessed via the National Air Quality Information System (https://sinca.mma.gob.cl). The chart differentiates between validated (blue), preliminary (orange), and unvalidated (green) records.*

*Table S1. Averages of particulate matter (MP) and CO recordings during the study period (June 15 - September 30, 2021; southern hemisphere) and annually.*

| | Study period | Annually |
|---|---|---|
| PM2.5 [ug/m3] | 48.8 ± 36.1 (CV: 74.0%) | 26.2 ± 30.6 (CV: 117%) |
| MP10 [ug/m3] | 56.9 ± 38.3 (CV: 67.3%) | 36.6 ± 30.8 (CV: 84.2%) |
| CO [ppm] | 0.78 ± 0.49 (CV: 61.5%) | 0.44 ±0.43 (CV: 97.7%) |

**Supplementary Document S4**

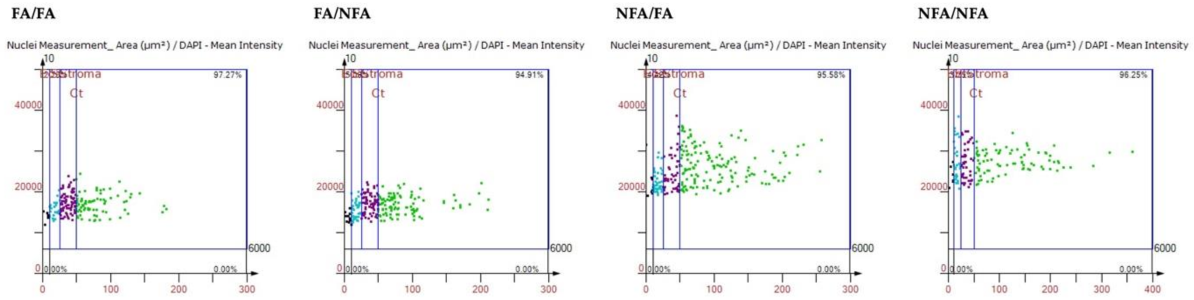


*Figure S4. Nuclear area analysis in placental tissue from pregnant rats exposed to fine particulate matter (PM2.5), performed using StrataQuest software. Nuclei were segmented and classified based on nuclear area, distinguishing three cell populations: cytotrophoblasts (Ct; in green), defined by nuclei with an area equal to or greater than 50 µm²; syncytiotrophoblasts (St; in purple), characterized by nuclei with an area between 25 µm² and 49.9999 µm²; and stromal endothelial cells (stroma; in light blue), identified by nuclei with an area between 10 µm² and 24.9999 µm². Results are shown for the four experimental groups: FA/FA (G2 rats reared and gestated in air-filtered chambers), FA/NFA (G2 rats reared in filtered chambers and gestated in non-filtered chambers), NFA/FA (G2 rats reared in non-filtered chambers and gestated in filtered chambers), and NFA/NFA (G2 rats reared and gestated in non-filtered chambers). The distribution and proportion of each nuclear population varied according to the exposure history to PM2.5 during postnatal development and gestation.*

## Supplementary Document S5

*Table S2. Area and intensity of GLUT1 and SVCT2 in cytotrophoblasts (CT), syncytiotrophoblasts (ST) and endothelial cells (EC).*

| | | Grupo | FA/FA | FA/NFA | NFA/FA | NFA/NFA | p-value |
|---|---|---|---|---|---|---|---|
| GLUT1 | CT | Area (um$^2$) | 314.6± 141.4 [ab] | 251.2±139.2 [a] | 272.7± 144.6 [b] | 304.3±169.1 | <0.0001 |
| | | Intensity | 6667±2127 [abc] | 5584±2256 [a] | 7516±2579 [b] | 8328±3485 [c] | <0.0001 |
| | ST | Area (um$^2$) | 245.9±129.6 [ab] | 187.3±106.5 [a] | 229.7±119.4 | 221.1±135.9 [b] | <0.0001 |
| | | Intensity | 6390±2379 [abc] | 5659±2450 [a] | 7633±2941 [b] | 7213±3536 [c] | <0.0001 |
| | EC | Area (um$^2$) | 217.3±120.5 [ab] | 135.3±101.1 [a] | 152.1±109.7 [b] | 189.2±114.7 | <0.0001 |
| | | Intensity | 6549±2337 [a] | 4764±2635 [a] | 7206±3556 | 7481±3479 | <0.0001 |
| SVCT2 | CT | Area (um$^2$) | 257.3±157.0 [a] | 154.8±124.4 [a] | 264.9±193.0 | 250.4±175.1 | <0.0001 |
| | | Intensity | 12485 ±2169 [ab] | 13128±2596 | 21637±2866 [a] | 26819±4852 [b] | <0.0001 |
| | ST | Area (um$^2$) | 195.0±144.9 [a] | 117.8±97.6 [a] | 218.8±161.9 | 192.5±142.5 | <0.0001 |
| | | Intensity | 12237±2305 [abc] | 13619±2961 [a] | 21367±3326 [b] | 26349±4784 [c] | <0.0001 |
| | EC | Area (um$^2$) | 166.8±126.9 [a] | 88.38±88.58 [a] | 156.3±130.7 | 171.6±140.8 | <0.0001 |
| | | Intensity | 12376±2566 [ab] | 13321±3165 | 22087±3866 [a] | 26812±5166 [b] | <0.0001 |

Identical letters indicate differences between groups.
